# Potential of Legume–Brassica Intercrops for Forage Production and Green Manure: Encouragements from a Temperate Southeast European Environment

**DOI:** 10.3389/fpls.2017.00312

**Published:** 2017-03-07

**Authors:** Ana M. Jeromela, Aleksandar M. Mikić, Svetlana Vujić, Branko Ćupina, Đorđe Krstić, Aleksandra Dimitrijević, Sanja Vasiljević, Vojislav Mihailović, Sandra Cvejić, Dragana Miladinović

**Affiliations:** ^1^Oil Crops Department, Institute of Field and Vegetable CropsNovi Sad, Serbia; ^2^Forage Crops Department, Institute of Field and Vegetable CropsNovi Sad, Serbia; ^3^Department of Field and Vegetable Crops, Faculty of Agriculture, University of Novi SadNovi Sad, Serbia; ^4^Biotechnology Department, Institute of Field and Vegetable CropsNovi Sad, Serbia

**Keywords:** aboveground biomass nitrogen yield, annual legumes, brassicas, forage dry matter yield, intercropping, land equivalent ratio

## Abstract

Legumes and brassicas have much in common: importance in agricultural history, rich biodiversity, numerous forms of use, high adaptability to diverse farming designs, and various non-food applications. Rare available resources demonstrate intercropping legumes and brassicas as beneficial to both, especially for the latter, profiting from better nitrogen nutrition. Our team aimed at designing a scheme of the intercrops of autumn- and spring-sown annual legumes with brassicas for ruminant feeding and green manure, and has carried out a set of field trials in a temperate Southeast European environment and during the past decade, aimed at assessing their potential for yields of forage dry matter and aboveground biomass nitrogen and their economic reliability via land equivalent ratio. This review provides a cross-view of the most important deliverables of our applied research, including eight annual legume crops and six brassica species, demonstrating that nearly all the intercrops were economically reliable, as well as that those involving hairy vetch, Hungarian vetch, Narbonne vetch and pea on one side, and fodder kale and rapeseed on the other, were most productive in both manners. Feeling encouraged that this pioneering study may stimulate similar analyses in other environments and that intercropping annual legume and brassicas may play a large-scale role in diverse cropping systems, our team is heading a detailed examination of various extended research.

## Legumes vs. Brassicas or Legumes and Brassicas?

With more than 700 genera and about 20,000 species, legumes (Fabaceae Lindl., syn. Leguminosae Juss.) are considered the third richest plant family (Lewis, 2005). They are renowned for having a remarkable place in global biodiversity, with countless members that are currently wild or semi-domesticated, but with great potential for cultivation. Legumes also provide diverse environment-friendly services, especially in the form of restoring, maintaining or enriching soil fertility through symbiotic nitrogen fixation. Due to their versatile nature, they play a manifold role in various food and feed production systems as the main, second, cover or cash crop, easily fitting in contrasting and complex agronomic patterns. In numerous regions throughout the world, legumes are staple crops (Vaz Patto et al., 2015), providing local human population and animals with everyday protein-rich diets (Gepts et al., 2005). All these facts endorse legumes with a flattering position of one of the most pivotal components of contemporary and future sustainable agriculture designs (Rubiales and Mikić, 2015).

Although comprising a significantly lesser number of around 370 genera and nearly 4,100 species of mainly herbaceous annuals, biennials and perennials, warm season shrubs and trees (Bremer et al., 2009), Brassicaceae Burnett (syn. Cruciferae Juss.) is a plant family that is by all means not poorer than legumes in economically important crops. Its most remarkable members include rapeseed (*Brassica napus* L.), with its globally widespread cultivar type with low content of erucic acid named *canola* (Lin et al., 2013), cabbage in both broad and narrow sense and with numerous conspecific taxa or variety groups (*Brassica oleracea* L.), and several species of mustards (*Brassica* spp. and *Sinapis* spp.). This family is also a home of thale cress (*Arabidopsis thaliana* L.), with a doubtless historical significance for comparative genetics and genomics and synteny research, being the first broad-scale studied and the most significant model plant species (Meinke et al., 1998).

Legumes and brassicas have much in common. Both comprise the most ancient domesticated plants in the world, such as chickpea (*Cicer arietinum* L.), lentil (*Lens culinaris* Medik.), pea (*Pisum sativum* L.) or bitter vetch [*Vicia ervilia* (L.) Willd.], *Brassica rapa*, and few *Sinapis* species (Zohary et al., 2012). They also share a characteristic of being not easily preserved as cereals and thus representing demanding archaeobotanical material: high content of protein in legumes and high content of oil in brassicas lead to a much higher degree of degradability in comparison to their fellow starch-rich crops belonging to the family Poaceae Barnhart and an impression that they were drastically less used by the humans in the past. One of the best evidenced findings related to brassicas comes from the famous Neolithic city of Çatalhöyük East, in Asia Minor, where mustards dated at 6,200–6,600 BC were used for ordinary use as oil and spice and for religious ceremonies 8,500 years ago (Fairbairn et al., 2007), along with cereals, grain legumes, and other plants. Apart from these links from deep past, legumes and brassicas today share a vast number of agricultural, economic, and social features. Both are widely present in many local wild floras and offer abundant gene pools for improving the existing and introducing novel cultivated species. Legumes and brassicas are highly adaptable to diverse cropping systems, due to the existence of autumn- and spring-sown cultivars and wide amplitudes of growing season length. They also comprise numerous multi-purpose crops, cultivated for grain, forage, green manure and are used as in human diets or animal feeding, non-food and fuel industry, pharmacy and medicine and ornamental purposes (Mikić et al., 2016).

The presented facts are our answer to the question from the title of this section. It has often been posed as a consequence of very short-term and solely profit-driven motifs, preferring, for instance, canola to pea and leading to a conclusion that growing one excludes the other or that they always compete for the same field. Rejecting what we deem an artificial antagonism, we declare: “Not legumes *or* brassicas, but legumes *and* brassicas” or, moreover, “legumes *with* brassicas.” This was the basis of the research we have been carrying out throughout the past decade and the results are presented in the forthcoming paragraphs.

## Together in Feeding Ruminants and Soil

A number of studies on intercropping demonstrate its positive effects on crop productivity, weed, pest, and disease control, yield stability, increased resource availability and exploitation, reflecting a renewed interest in this ancient practice. This interest arose from a growing awareness of the environmental problems in modern agriculture associated with excessive use of synthetic fertilisers and pesticides. Due to legumes ability of nitrogen fixation, niche complementarity and positive legumes–cereals interactions, such intercropping systems are known to produce increase in biomass relative to monoculture, a phenomenon described as overyielding (Hector et al., 1999; Beckage and Gross, 2006). Li et al. (2007) showed that faba bean can facilitate mobilisation and uptake of deficient phosphorus by cereals resulting in overyielding. This beneficial interspecific interaction (facilitation) can be based on complementary and more complete sharing and exploitation of other resources, such as solar radiation, water, and soil (Brooker et al., 2015).

In comparison to the accumulated knowledge and available literary resources related to the intercrops of legumes and cereals, it may regretfully be noticed that very little is known on both basic and applied aspects of the mixtures of legumes and brassicas (Mikić et al., 2012). The potential of legume–brassica intercropping has been investigated for various purposes in Europe, Asia, and North America, reflecting the endeavours of researchers to address problems and meet the farmers’ needs specific for these different agro-environments. In France, Sweden, and Canada, the weeds in oilseed rape are tackled by intercropping frost-sensitive legume as living mulch (Bergkvist, 2003; Thériault et al., 2009; Cadoux et al., 2015; Lorin et al., 2015). The advantage of intercropping annual legumes with cabbage and cauliflower in Turkey (Yildirim and Guvenc, 2005, 2006) and broccoli in the USA (Coolman and Hoyt, 1993) over sole crops, were demonstrated in vegetable production. In India, intercropping mustard and annual legumes for oil and food, increases yield stability, nutrient availability, and water use efficiency and provides monetary advantage over sole crops (Banik et al., 2000; Singh et al., 2010; Devi et al., 2014). In several studies, dealing mostly with soil nutrition aspects, it was commonly assessed that a brassica component may profit from being intercropped with a legume. In a series of rhizotron trials, including the intercrops of faba bean (*Vicia faba* L.) with rapeseed and common vetch (*Vicia sativa* L.) with cabbage, it was found out that, if intercropped, the ramification distribution along the taproot was different than in pure stands, which reduced the competition, as well as that the transfer of nitrogen from legumes to brassicas was significant (Cortés-Mora et al., 2010). Also, the presence of the legumes is demonstrated to be able to help reducing nitrogen fertiliser input (Jamont et al., 2013). A vast and complex field trial in Saskatchewan, Canada, brought forth the thorough claims that intercropping canola with pea improved seed yield, nitrogen uptake and net returns, recommending it as one of the most promising intercrop designs for organic farming systems (Malhi, 2012).

In Southeast Europe, legume–other crop intercropping, especially, legumes–cereals intercropping system has a long tradition for forage production for cattle feed, namely cows. A constant farmers’ demand for high yield and good quality forage that will provide high and good quality milk production, prompted research on various combinations of legume–non-legume intercropping patterns, including legume–brassica intercrops. Institute of Field and Vegetable Crops, Novi Sad, Serbia and the Department of Field and Vegetable Crops of the Faculty of Agriculture of the University in Novi Sad, Serbia established a concerted set of field trials, designed jointly by the teams of both institutions and carried out at the Experiment Field at Rimski Šančevi in the vicinity of Novi Sad, on a carbonated chernozem soil and in various consecutive seasons during the past 10 years. The examined species included the following annual legumes and brassicas: common vetch (autumn- and spring-sown), grass pea (*Lathyrus sativus* L.), hairy vetch (*Vicia villosa* Roth), Hungarian vetch (*Vicia pannonica* Crantz), Narbonne vetch (*Vicia narbonensis* L.), pea (autumn- and spring-sown), brown mustard [*Brassica juncea* (L.) Czern.], field mustard [*B. rapa* ssp. *oleifera* (DC.) Metzg.], fodder kale (*B. oleracea* L. var. *viridis* L.), rapeseed (autumn- and spring-sown) and white mustard (*Sinapis alba* L.). These species are commonly used for forage and green manure in the Southeast Europe and were chosen for testing various intercrop combinations.

**FIGURE 1:**
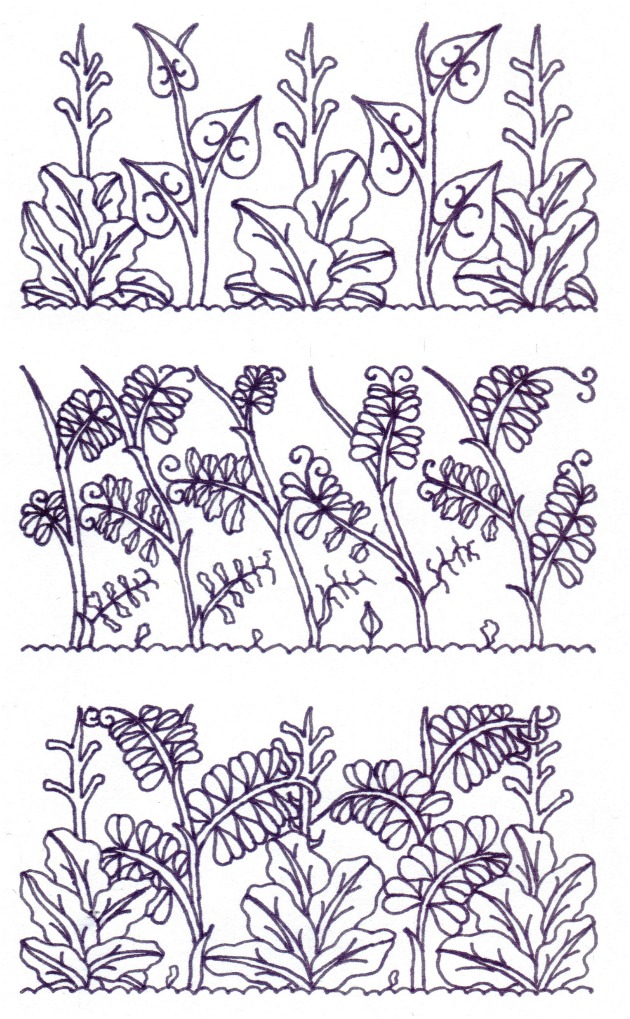
**A scheme of intercropping legumes with brassicas for forage production and green manure: *Top row*—often sown in wide rows, *Brassicas* suffer from heavy weed infestations; *Middle row*—forage legumes easily fight the weeds, but are quite prone to lodging, with an outcome in partial or complete withering and loss of lower leaves; *Bottom row*—intercropping legumes and brassicas is beneficial for both, providing legumes with mechanical support and assisting brassicas to bring forth its full potential**.

Our research was founded upon a scheme specifically developed for intercropping autumn- and spring-sown annual legumes and brassicas for both forage production and green manure, in other words, immature aboveground biomass cultivation, that is in bloom and not in full grain maturity (Marjanović-Jeromela et al., 2015c). The scheme is a result of long-term observations of pure stands of various annual legumes and brassicas and consists of three segments (**Figure 1**).

1. A prominently profuse growth of aboveground biomass in forage annual legume cultivars, essential for obtaining high yields of both forage dry matter and aboveground biomass nitrogen, is simultaneously and rather often their main disadvantage: with an insufficient mutual support with tendrils, they are prone to lodging before reaching the stage of full bloom, already lose a significant number of primarily developed leaves and suffer from extreme shade and ideal conditions for disease development in the lower half of the stand (**Figure 1**, top row).

2. On the other hand, brassicas are usually sown at a wider row spacing in order to provide an appropriate space for free and plentiful leaf growth development: however, this is often an opportunity for heavy weed infestations almost immediately after the brassica crop emergence, ending with high proportions of undesirable plants in total aboveground biomass yield and poorer brassica forage quality (**Figure 1**, middle row).

3. If intercropped with each other, forage annual legumes and brassicas have multiple benefits: brassica serves as an additional and essential support for annual legume, resulting in an improved standing ability, preserved photosynthesis-active leaves and enhanced dry matter production and higher forage protein yield, while legume serves as a powerful weed competitor on behalf of brassica and assists it in accumulating nitrogen in its aboveground biomass (**Figure 1**, bottom row).

This scheme may be compared to those developed for intercropping various annual legumes with each other or pea cultivars with different leaf types, where those, such as white lupin (*Lupinus albus* L.) or faba bean and semi-leafless pea act as supporting crops for forage field pea or vetches (*Vicia* spp.) and normal-leafed pea, respectively (Mikić et al., 2015b).

All the genotypes of both annual legumes and brassicas for intercropping were selected according to the same sowing season, namely autumn and spring, and the concurrent time of cutting, that is, full flower in annual legumes and budding in brassicas, as the optimum moment for both forage production and green manure cultivation. The terms we use, *forage dry matter* and *aboveground biomass* (immature, that is, in bloom and not in full grain maturity) are in fact synonyms: the former denotes that it is cut, collected and used for ruminant feeding, while the latter designs its use as incorporated green manure with potential for increasing soil nitrogen content. These rules are also used in adequately choosing the genotypes for establishing mutual legume mixtures (Ćupina et al., 2011b). In all the examined legume–brassica intercrops, the sowing rates of both components were reduced by half in comparison to their pure stands, as is also present in the designs of brassica–cereal and other crop mixtures (Marjanović-Jeromela et al., 2015b; Mihailović et al., 2015b). A careful choice and adjustment of sowing machine enabled joint sowing of legumes and brassicas, thus avoiding double sowing. Comparing to some other systems, such as relayed intercropping, which require inter-row sowing and temporally separated sowing and harvest, these concurrent mechanised operations are less labour demanding and the economic benefits they could bring may stimulate agriculture machinery manufacturers to develop the machinery adapted for intercropping. Such practice, in case that legume–brassica intercrop schemes are considered for wide production, may serve as another strong encouragement for the farmers to embrace it.

The sole crops of both autumn- and spring-sown annual legumes and brassicas in our set of trials confirmed their high potential for forage production and green manure in temperate regions (Ćupina et al., 2011a; Mihailović et al., 2015a). Legumes had the average values of both forage dry matter yield and aboveground biomass nitrogen in comparison to those of brassicas (**Table 1**; Ćupina et al., 2010; Krstić et al., 2011; Antanasović et al., 2012). In the case of forage dry matter, they ranged from 5.9 t ha^-1^ in Narbonne vetch to 9.6 t ha^-1^ in hairy vetch, and between 3.7 t ha^-1^ in brown mustard and 7.5 t ha^-1^ in fodder kale. Regarding aboveground biomass nitrogen yield, it varied between 171 kg ha^-1^ in Narbonne vetch and 327 kg ha^-1^ in grass pea and from 103 kg ha^-1^ in brown mustard to 199 kg ha^-1^ in fodder kale (Ćupina et al., 2013; Mikić et al., 2013). The difference between the trends in forage dry matter yield and aboveground biomass nitrogen yield exist due to the difference in the proportion of crude protein or, in other words, nitrogen in individual species (Ćupina et al., 2014) and is equivalent to those observed in the similar trials with the autumn- and spring-sown intercrops of brassicas and cereals (Mihailović et al., 2014).

**Table 1:** Average values of forage dry matter yield (t ha^-1^), aboveground nitrogen yield (kg ha^-1^), and their land equivalent ratios (LER_FDMY_ and LER_AGBY_) in a set of trials at Rimski Šančevi from 2005 to 2014 (Ćupina et al., 2013; Mikić et al., 2013, 2015a; Marjanović-Jeromela et al., 2015a).

Season	Pure stand/intercrop	Total forage dry matter yield	LER_FDMY_	Total aboveground nitrogen yield	LER_AGBY_
Autumn	Common vetch	8.5	–	258	–


	Hairy vetch	9.6	–	307	–


	Hungarian vetch	6.5	–	214	–


	Pea	9.2	–	275	–


	Field mustard	6.5	–	188	–


	Fodder kale	7.5	–	199	–


	Rapeseed	6.9	–	189	–


	Common vetch + field mustard	9.9	1.21	282	1.07


	Common vetch + fodder kale	8.4	1.05	238	1.05


	Common vetch + rapeseed	8.3	1.08	214	0.97
	Hairy vetch + field mustard	9.3	1.11	379	1.02
	Hairy vetch + fodder kale	9.3	1.06	303	1.13
	Hairy vetch + rapeseed	9.4	1.09	319	1.18
	Hungarian vetch + field mustard	9.3	1.25	224	1.05
	Hungarian vetch + fodder kale	8.0	1.14	198	0.96
	Hungarian vetch + rapeseed	7.4	1.10	162	0.81
	Pea + field mustard	10.5	1.18	272	1.10
	Pea + fodder kale	8.8	1.06	254	1.07
	Pea + rapeseed	8.9	1.10	242	1.03
	LSD_0.05_	0.8	0.03	31	0.07
Spring	Common vetch	7.6	–	217	–
	Grass pea	8.8	–	327	–
	Narbonne vetch	5.9	–	171	–
	Pea	8.4	–	240	–
	Brown mustard	3.7	–	103	–
	Rapeseed	6.9	–	171	–
	White mustard	4.2	–	189	–
	Common vetch + brown mustard	6.6	1.11	206	1.14
	Common vetch + rapeseed	8.1	1.12	238	1.23
	Common vetch + white mustard	6.9	1.20	216	1.04
	Grass pea + brown mustard	8.4	1.02	404	1.01
	Grass pea + rapeseed	8.6	1.07	302	1.16
	Grass pea + white mustard	8.0	1.13	352	1.18
	Narbonne vetch + brown mustard	6.6	1.25	198	1.28
	Narbonne vetch + rapeseed	6.5	1.21	199	1.16
	Narbonne vetch + white mustard	6.3	1.23	178	1.00
	Pea + brown mustard	8.0	1.18	249	1.18
	Pea + rapeseed	8.6	1.12	254	1.23
	Pea + white mustard	8.0	1.25	245	1.09
	LSD_0.05_	0.8	0.09	44	0.06

*LSD_*0.05*_ = Least significant difference at *p* ≤ 0.05*.

The analysis of the average values of forage dry matter yield in the carried out number of field trials show that the autumn-sown intercrops of annual legumes and brassicas were superior to the spring-sown ones, with a variation from 7.4 t ha^-1^ in the intercrop of Hungarian vetch with rapeseed to 10.5 t ha^-1^ in the intercrop of pea with field mustard and from 6.3 t ha^-1^ in the intercrop of Narbonne vetch and white mustard to 8.6 t ha^-1^ in the intercrops of both grass pea with rapeseed and pea with rapeseed, respectively (**Table 1**). The intercrops of hairy vetch were most productive in the autumn-sown group, with all three mixtures having forage dry matter yield higher than 9 t ha^-1^ (Ćupina et al., 2013), while, among the spring-sown intercrops, those of grass pea and pea were productive than the other two, both having forage dry matter yield not lower than 8.0 t ha^-1^ (Mikić et al., 2013).

The results related to the average values of aboveground biomass nitrogen yield were generally characterised by a similar trend to that of forage dry matter yield, but more in the autumn-sown group than the spring-sown one: within the former, it was the intercrops of hairy vetch that also had the highest green manure potential, with more than 379 kg ha^-1^ in all three cases, but, within the latter, it was grass pea that was the most productive, with also more than 300 kg ha^-1^ (**Table 1**). This could be, at least partially, explained by a rather great proportion of grass pea in the total aboveground biomass and thus contributing more to the average aboveground biomass nitrogen yield, with the data on these parameters too broad to be shown in this review. The range of average aboveground biomass nitrogen yield among the autumn-sown intercrops was from 162 kg ha^-1^ in the intercrop of Hungarian vetch with rapeseed to 379 kg ha^-1^ in the intercrop of hairy vetch with field mustard (Marjanović-Jeromela et al., 2015a), while among the spring-sown intercrops it was from 178 kg ha^-1^ in the intercrop of Narbonne vetch with rapeseed to 404 kg ha^-1^ in the intercrop of grass pea with brown mustard (Mikić et al., 2015a).

The land equivalent ratio (LER) is a widely used relative indicator of economic reliability of an intercrop, unlike yield as an absolute one. It is calculated on the basis of the yield of each component in an intercrop and in its pure stand; if surpassing 1.00, an intercrop is considered economically reliable (Mikić et al., 2015b). An overview of the accumulated results related to the LER values of both forage dry matter yield (LER_FDMY_) and aboveground biomass nitrogen yield (LER_AGBY_) shows that all the intercrops had higher LER_FDMY_ than 1.00, as well as that a very few intercrops had LER_AGBY_ values lower than 1.00 (**Table 1**). The intercrops of Hungarian vetch with field mustard, Narbonne vetch with brown mustard and pea with white mustard had the highest average values of LER_FDMY_ (Ćupina et al., 2013), 1.25 in all three, while the intercrop of Narbonne vetch with brown mustard had the highest average value of LER_AGBY_, 1.28. Among the autumn-sown intercrops, the highest values of LER_FDMY_ and LER_AGBY_ were in those of Hungarian vetch and hairy vetch, respectively (Mikić et al., 2015a), while among the spring-sown intercrops, the highest values of LER_FDMY_ and LER_AGBY_ were in those of Narbonne vetch (Mikić et al., 2013) and pea (Marjanović-Jeromela et al., 2015a).

### Legume + Brassica Perspectives

The authors are fully aware of a multitude of issues stemming out from the achieved, accumulated and presented results and all the arguments possibly raised by a reader. However, all of us being practical agronomists and breeders, we aimed first at firmly demonstrating that our schemes for intercropping annual legume and brassicas answer the most important demand by the farmers: that the forage yield is higher than in pure stands. Having a positive answer, along with the good results in relation to the economic reliability, we feel encouraged to proceed with a detailed examination of various extended research, such as assessing the intercrop chemical composition, examining the relationship towards various forms of abiotic and biotic stress and, especially, studying the complex underground aspects of nutrition and allelopathy. We remain convinced that this pioneering review may stimulate similar analyses in other environments, as well as that intercropping annual legume and brassicas may secure its place in diverse farming systems and on a larger scale based on its many advantages, not least the use of local legumes and *Brassica* species, thus strengthening local economies as well as promoting conservation, since no fertilisers are required.

## Author Contributions

AJ, AM, BĆ, and VM planned and designed the experiments; AJ, AM, ĐK, AD, and SC: Performed the research; AJ, AM, BĆ, VM, SV, and DM contributed to interpretation and analysis of results; AJ, AM, SV, BĆ, ĐK, AD, SVa, VM, SC, and DM wrote and approved the manuscript.

## Conflict of Interest Statement

The authors declare that the research was conducted in the absence of any commercial or financial relationships that could be construed as a potential conflict of interest.
